# New Insight in HDACs: Potential Therapeutic Targets for the Treatment of Atherosclerosis

**DOI:** 10.3389/fphar.2022.863677

**Published:** 2022-04-21

**Authors:** Yi Luan, Hui Liu, Ying Luan, Yang Yang, Jing Yang, Kai-Di Ren

**Affiliations:** ^1^ Research Center for Clinical System Biology, Translational Medicine Center, the First Affiliated Hospital of Zhengzhou University, Zhengzhou, China; ^2^ School of Laboratory Medicine, Xinxiang Medical University, Xinxiang, China; ^3^ Henan Key Laboratory of Precision Clinical Pharmacy, Zhengzhou University, Zhengzhou, China; ^4^ Department of Pharmacy, the First Affiliated Hospital of Zhengzhou University, Zhengzhou, China; ^5^ Department of Physiology and Neurobiology, School of Basic Medical Sciences, Zhengzhou University, Zhengzhou, China

**Keywords:** atherosclerosis (AS), histone deacetylases (HDACs), vascular systems, HDAC inhibitors, deacetylation

## Abstract

Atherosclerosis (AS) features include progressive hardening and reduced elasticity of arteries. AS is the leading cause of morbidity and mortality. An increasing amount of evidence showed that epigenetic modifications on genes serve are a main cause of several diseases, including AS. Histone deacetylases (HDACs) promote the deacetylation at lysine residues, thereby condensing the chromatin structures and further inhibiting the transcription of downstream genes. HDACs widely affect various physiological and pathological processes through transcriptional regulation or deacetylation of other non-histone proteins. In recent years, the role of HDACs in vascular systems has been revealed, and their effects on atherosclerosis have been widely reported. In this review, we discuss the members of HDACs in vascular systems, determine the diverse roles of HDACs in AS, and reveal the effects of HDAC inhibitors on AS progression. We provide new insights into the potential of HDAC inhibitors as drugs for AS treatment.

## Introduction

As a critical potential pathology of cardiovascular disease (CVD), atherosclerosis (AS) is characterized by the progressive hardening and reduced elasticity of arteries; AS is a leading cause of morbidity and mortality (Aghamajidi et al., 2021). Its progression will ultimately lead to myocardial infraction, ischemic stroke, cerebrovascular incidents, and peripheral vascular disease, thereby increasing the risk of death (Yang et al., 2021). The most outstanding feature of AS is the plaque formation in the arteries. Vascular cell homeostasis low-density lipoprotein (LDL) oxidation, monocyte recruitment, macrophage-derived foam cell formation, and thrombus formation play important roles during AS progression (Luan et al., 2021). Among them, vascular homeostasis is one of the major AS risk factors (Rajendran et al., 2013). The maintenance of vascular homeostasis requires the joint participation of various vascular cells. Vascular cells are composed of endothelial cells (ECs) and smooth muscle cells (SMCs). The alteration of proliferation, migration, and apoptosis of ECs and SMCs is indispensable in AS (Dai et al., 2018). Endothelial dysfunction includes abnormal proliferation, migration, and apoptosis and contributes to enhanced endothelial permeability to lipoproteins, increased leucocyte migration and adhesion, and reduced nitric oxide production, thereby subsequently triggering fatty streak formation (Liu et al., 2007). The proliferation and migration of SMCs are critical in the formation of fatty steak, which induces advanced lesions and fibrous cap formation (Clarke et al., 2006). AS is very likely to form at certain areas of arteries, such as branching points and bends, because of the local disturbance of endothelial functions (Ravensbergen et al., 1998). In addition, lipid metabolism disorder also plays an important role in AS progression. As reported, LDL, especially that modified by oxidation, enzymatic processing, desialylation, and aggregation, is the main substance in the atherosclerotic lesions (Torzewski, 2021). These LDL modifications are prone to induce immune response; thus, the body forms highly atherogenic circulating LDL, which are involved in immune complexes (Zhang et al., 2018a). Other cell types are also involved in AS pathogenesis, such as macrophages and stem cells (Wang et al., 2015a; Takamura et al., 2017; Ruytinx et al., 2018; Kloc et al., 2020).

An increasing amount of evidence indicated that epigenetic modifications on genes are the main cause of many diseases, such as, cancer, and CVDs (Yang et al., 2021). Epigenetic modifications can modulate gene expression without altering gene sequences, thereby facilitating rapid and reversal of the regulation of targeted genes (Wee et al., 2014). Epigenetic modifications are composed of DNA and histone modifications. DNA modifications are inheritable, whereas histone modifications are not (Qin et al., 2021). Histone methylation and acetylation are the main forms of histone modification (Rajan et al., 2020). Acetylation changes the condensation of chromatin and has been considered as a therapeutic target. Histone acetylation is tightly controlled by histone acetyltransferases (HATs) and histone deacetylases (HDACs), which exert contradictory functions (Raman and Rai, 2018). Acetylation in histones and non-histones can be catalyzed by HATs and removed by HDACs. In most cases, HDACs repress gene expressions through interactions with histones and transcription factors (Gallinari et al., 2007).

### The Classification of HDACs

**FIGURE 1:**
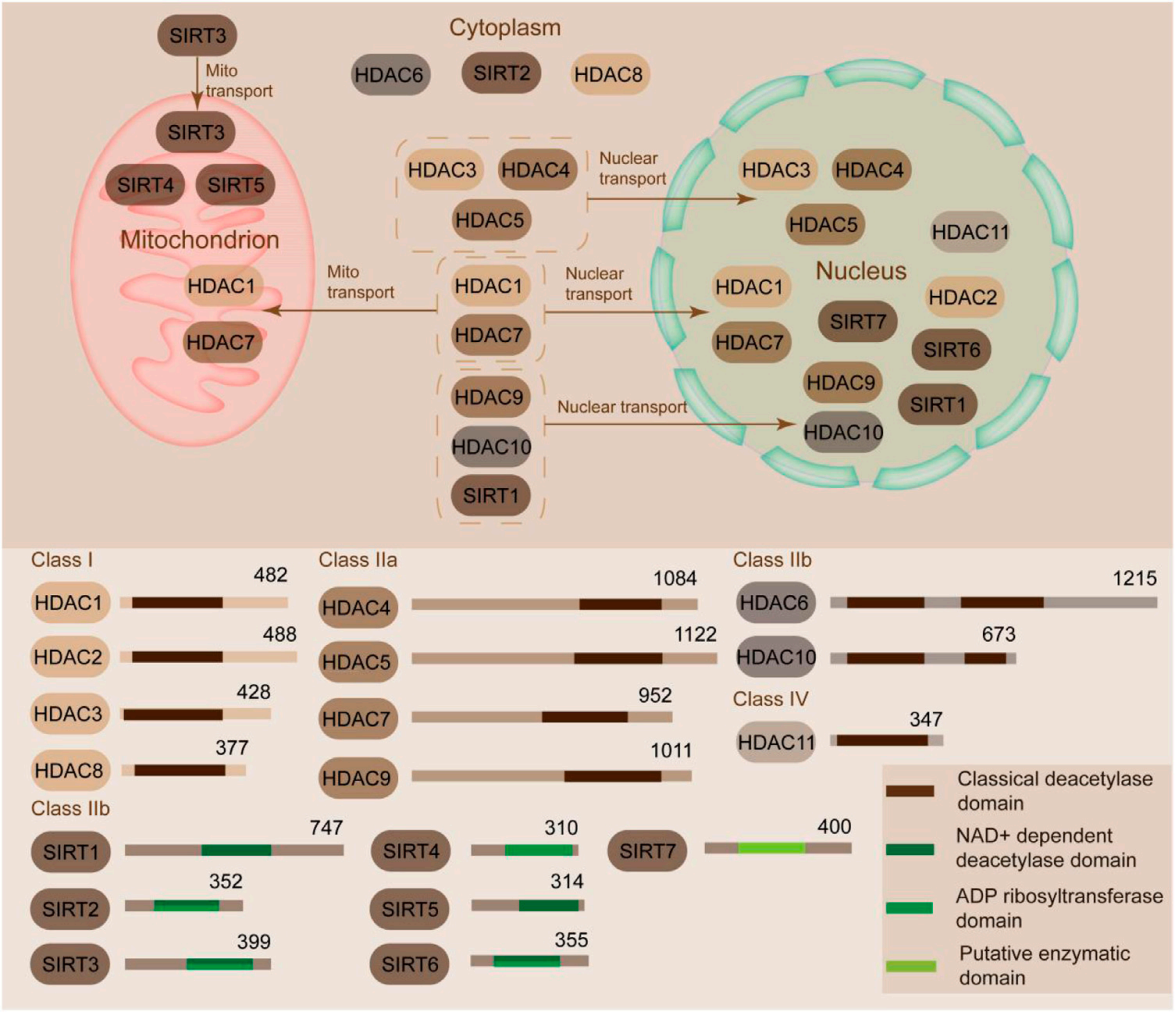
Classification and sublocation of HDACs. HDACs can be classified into Class I, II, III, and IV according to similarities. Class I HDACs (HDAC1/2/3/8) are mostly located in the nucleus. Class II HDACs are subclassified into subclass IIa (HDAC4, 5, 7 and 9) and subclass IIb (HDAC6 and 10). Class IIa HDACs translocate between cytoplasm and nucleus. Class IIb members mostly localize in the cytoplasm. Class III contains seven members (SIRT1 to 7). SIRT1/2 shuttle between the nucleus and cytoplasm. SIRT6/7 are mostly in the nucleus. SIRT3/4/5 are localized in the mitochondria. Class IV HDAC (HDAC11) is predominantly located in the nucleus.

HDACs promote the deacetylation at lysine residues and condense the chromatin structures, thereby further inhibiting the transcription of downstream genes (Walther et al., 2020). HDACs are composed of two families, namely, HDAC and sirtuin, which include 18 members (Dai et al., 2021). These members can be clearly classified into four groups, namely, Class I, II, III, and IV, based on the enzymatic activities, domain structures, functions, and sequence similarity (Figure 1) (Zhang et al., 2020). Class I HDACs (HDAC1/2/3/8) share high sequence similarity with yeast Rpd3 and are mostly located in the nucleus (Zaidi et al., 2020). Class II HDACs are subclassified into subclass IIa (HDAC4, 5, 7, and 9) and subclass IIb (HDAC6 and 10). Class IIa HDACs are specifically expressed in muscle and heart tissues; they translocate between cytoplasm and nucleus, interact with kinase proteins (calcium-independent protein kinases and the MAPK), and act as signal transducer (Ziegler et al., 2020). Class IIb members mostly localize in the cytoplasm and are distinguished from the class IIa members in terms of the tandem deacetylase domains (Yu et al., 2020). Class III is composed of the sirtuin family and contains seven members (SIRT1 to 7), which share high sequence similarity with the yeast protein silent information regulator 2 (Sir2) (Chen et al., 2020). These HDACs require NAD^+^ to catalyze the deacetylation reaction due to their conserved catalytic domain, whereas other HDACs rely on the binding of zinc molecule as an activator (Vaquero et al., 2007). SIRT1/2 shuttle between the nucleus and cytoplasm. SIRT6/7 are mostly in the nucleus, and SIRT3/4/5 are localized in the mitochondria (Figure 1) (Villalba and Alcaín, 2012). Class IV HDAC (HDAC11, the sole member in class IV) shares sequence homology with Class I and II members (Seto and Yoshida, 2014). HDAC11 modulates the protein stability of CDT1 and negatively modulates the expression of interleukin (IL)-10 and the activity of T cells, thereby indicating the potential role of HDAC11 in AS progression (Glozak and Seto, 2009). The classical HDAC family also consists of Class I, II, and IV besides Class III (Dai et al., 2021).

Although HDACs are commonly recognized as enzymes that catalyze the removal of acetyl group from histones, present studies have identified many other non-histone substrates, such as NF-kB, E2F1, SP1, KLF2/4, and STAT1 (Villagra et al., 2010). Considering the diversity of HDAC substrates, they are also related to multiple cellular processes and several diseases, including AS (Ke et al., 2021). Notably, the sirtuin family is widely acknowledged because of its diverse roles in vascular functions. In this review, we discuss the role of HDACs in vascular function and AS process and the pharmacological effects of HDAC inhibitors (HDACi) on AS treatment.

### HDACs in Modulating the Function of Endothelial Cells (ECs)

**FIGURE 2:**
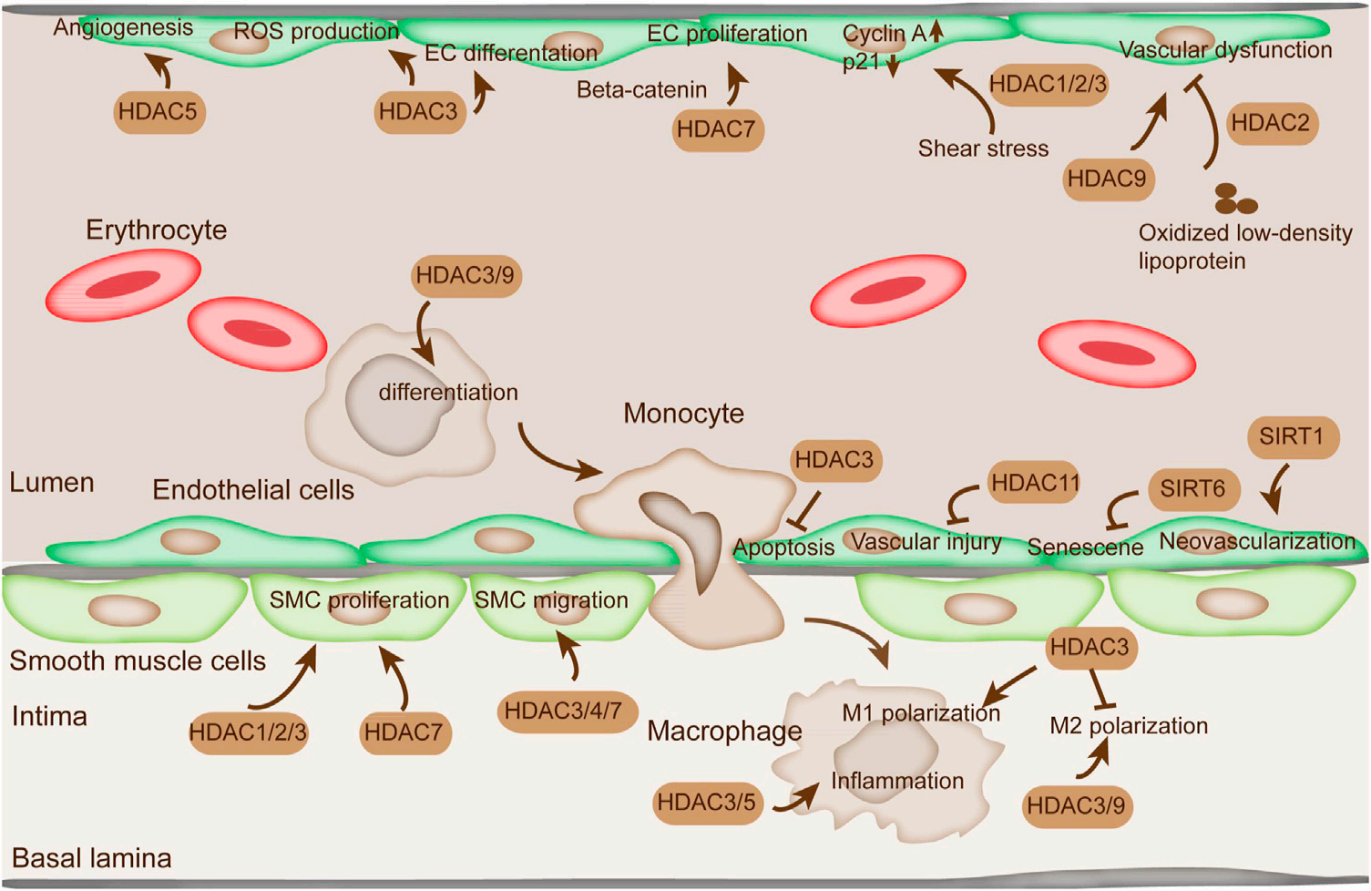
The function of HDACs in ECs, SMC, and macrophages. The alteration of proliferation, migration, and apoptosis of ECs and SMCs is indispensable in AS. HDAC1/2/3/7 regulate SMC proliferation. HDAC3/4/7 are involved in SMC migration. HDAC3/5 modulate inflammation in macrophage. HDAC3/9 regulate proinflammatory gene expression by modulating M2 macrophage polarization. HDAC11 plays a critical role in vascular injury. SIRT1 is directly related to EC senescence and apoptosis. SIRT6 protects EC from senescence. HDAC1/2/3 are reportedly involved in EC proliferation mediated by oscillatory shear stress. In addition, HDAC5 represses angiogenesis in ECs.

Endothelial cells (ECs) are a main type of cells in blood vessels that modulate vascular tone, blood coagulation, and mediate inflammatory reaction (Wautier and Wautier, 2021). HDACs are a major group of histone deacetylases that are extensively involved in endothelial cell function regulation (He et al., 2011). HDACs are critical in modulating the gene expressions involved in vascular homeostasis and vessel development as transcriptional cofactors (Figure 2) (Zhao et al., 2020). Among the HDACs, HDAC1/2/3 are reportedly involved in EC proliferation mediated by oscillatory shear stress, which increases the expression of cyclin A and decreases p21 (Bazou et al., 2016). HDAC2 overexpression suppresses vascular dysfunction induced by oxidized LDL (Pandey et al., 2014). HDAC3 mediates EC differentiation from embryonic stem cells and keeps endothelial integrity dependent on PI3K/Akt and TGFβ2 pathways (Wang et al., 2021). Moreover, the knockdown of HDAC3 is associated with the decrease of Nox4, a major source of reactive oxygen species (ROS) production in the vascular wall, thereby suggesting the critical role of HDAC3 in EC function (Fu et al., 2020).

Class IIa HDACs are involved in vascular homeostasis response to signals in the modulation of gene expression (Clocchiatti et al., 2011). For instance, HDAC4/5 nuclear translocation is initiated by nitric oxide (NO) to modulate the activation of protein phosphatase 2A (PP2A) (Illi et al., 2008). Moreover, NO promotes the formation of a complex containing HDAC3, HDAC4, HDAC5, and an active PP2A (Illi et al., 2008). HDAC5 represses angiogenesis by mediating the expression profile of angiogenic genes in ECs (Shiva Shankar and Willems, 2014). Its overexpression weakens sprout formation, whereas its inhibition by the inhibitors manifests pro-angiogenic effect by inducing EC migration, sprouting, and tube formation. Previous studies indicated that HDAC7 regulated EC proliferation by modulating beta-catenin translocation. HDAC7 overexpression prevented nucleus beta-catenin translocation and inhibited EC proliferation (Margariti et al., 2010). HDAC7 knockdown promoted the nuclear translocation of beta-catenin and inhibited the levels of cyclin D1, cyclin E1, and E2F2, thereby causing EC hypertrophy. Moreover, HDAC7-mediated EC proliferation suppression can be partially ameliorated by VEGF through the induction of HDAC7 degradation via PLCg-IP3K signaling pathway (Chang et al., 2006). Another HDAC, HDAC9, affected EC dysfunction and permeability dysfunction in oxygen-glucose deprivation-induced ischemia in the cerebral hemisphere (Shi et al., 2016).

The sirtuin family is also involved in mediating the function of ECs (Matsushima and Sadoshima, 2015). SIRT1 is critical in modulating neovascularization by regulating angiogenic ability when stimulated by angiogenic cues (Botti et al., 2014). The deacetylase activity of SIRT1 on *Foxo* transcription factors repressed its transcription and restrained EC proliferation, migration, and neovessel formation (Gu et al., 2016). SIRT6 protected EC from senescence. SIRT6 depletion aggravated the percentage of senescent cells in HUVEC and aortic endothelial cells and suppressed the formation of tubule networks (Cardus et al., 2013). The sole member of class IV, HDAC11, restored the expression of angiogenic factor in response to carotid artery ligation in mice (Nunez-Alvarez and Suelves, 2021). HDAC11 depletion mitigated vascular injury in mice, suggesting its critical role in vascular injury (Nunez-Alvarez and Suelves, 2021).

The apoptosis of ECs also plays critical role in endothelial dysfunction during AS progression (Qin et al., 2017). In addition, recent studies implied that apoptosis in luminal EC probably induced the formation of thrombus on eroded plaques without rupture (Quillard et al., 2017). HDACs are believed to affect EC apoptosis. Knockdown of HDAC3 induced extensive membrane blebs and more Annexin V staining and reduced cell survival (Lee and Chiu, 2019). In addition, HDAC3 overexpression facilitated Akt phosphorylation and activated its kinase activity (Long et al., 2017). Therefore, HDAC3 is crucial in maintaining cell survival and prevents AS by activating Akt. In addition, SIRT1 is directly related to EC senescence and apoptosis (Liu et al., 2018). Hou et al. showed that SIRT1 prevented the externalization of early membrane apoptotic phosphatidylserine, and the DNA degradation was dependent on Akt1 and FoxO3a (Hou et al., 2011). Other studies demonstrated that SIRT1 mediated EC proliferation and senescence by modulating a serine/threonine kinase and tumor suppressor LKB1 (Zu et al., 2010). Inhibition of HDACs by valproic acid induced phosphorylation of extracellular signal-regulated kinase1/2 (ERK 1/2) and subsequently caused phosphorylation of Bcl-2 and EC apoptosis inhibition in response to serum starvation (Joanna et al., 2009).

### HDAC Modulation in Smooth Muscle Cells (SMCs)

**FIGURE 3:**
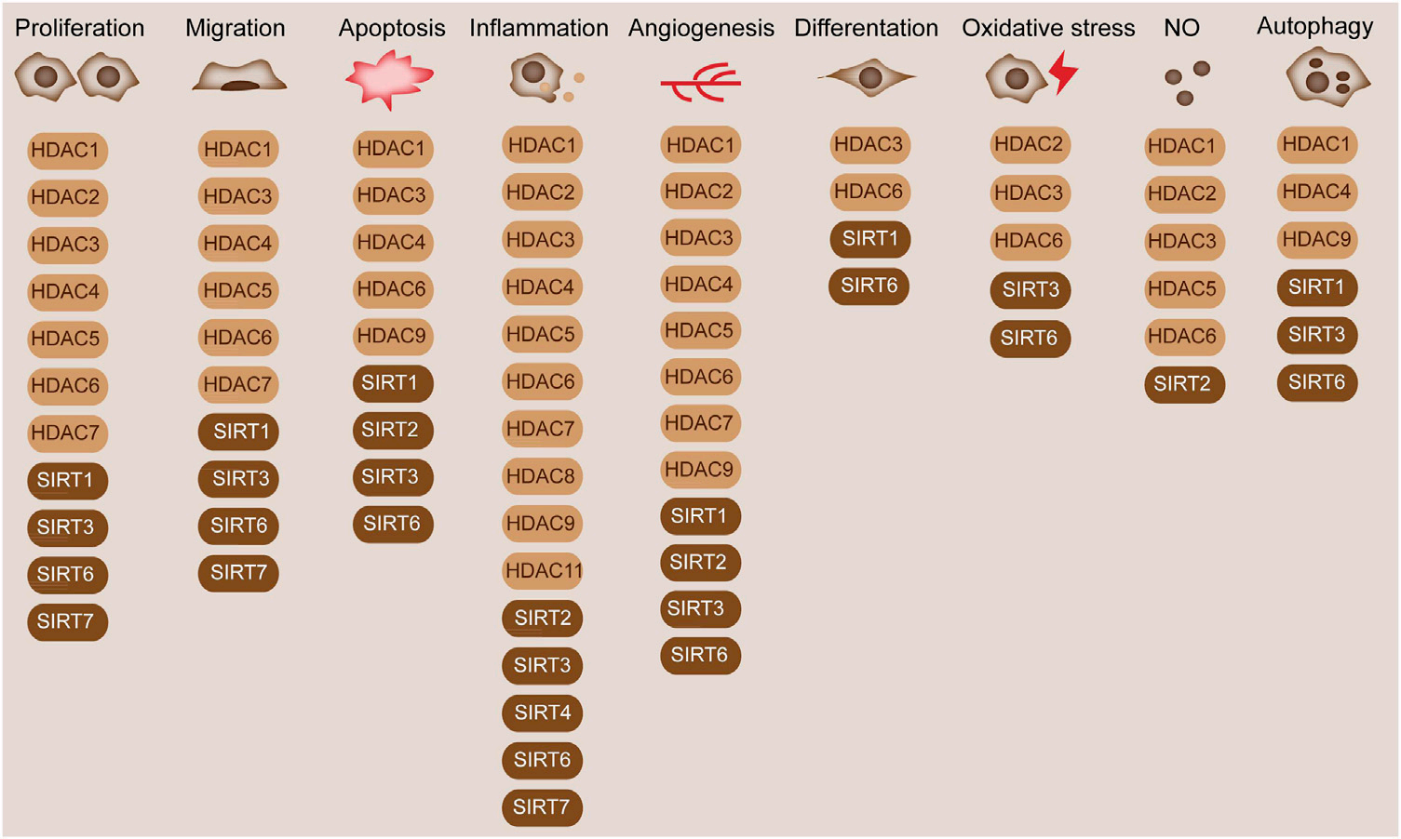
Diverse functions of HDACs in blood vessels. HDACs are widely involved in the cellular processes in blood vessels, such as cell proliferation, migration, and differentiation. HDAC1/2/3/4/5/6/7 and SIRT1/3/6/7 are involved in cell proliferation. HDAC1/3/4/5/6/7 and SIRT1/3/6/7 regulate cell migration. HDAC1/3/4/6/9 and SIRT1/2/3/6 mediate cell apoptosis. HDAC1/2/3/4/5/6/7/8/9/11 and SIRT2/3/4/6/7 modulate inflammation. HDAC1/2/3/4/5/6/7/9 and SIRT1/2/3/6 modulate angiogenesis. HDAC3/6 and SIRT1/6 modulate cell apoptosis. HDAC2/3/6 and SIRT3/6 are associated with oxidative stress. HDAC1/2/3/5/6 and SIRT2 are associated with NO production. HDAC1/4/9 and SIRT1/3/6 modulate cell autophagy.

The proliferation of smooth muscle cells (SMCs) is necessary in the formation of neointima and arteriosclerosis (Daniel and Sedding, 2011). Following EC injury and activation, diverse growth factors (e.g., PDGF and TGF-beta) and cytokines (interferon-ɣ) were released and promote SMC proliferation, which aggravated the generation of advanced lesions during AS (Annoni et al., 1992). Interference in the level of HDAC1/2/3 attenuated SMC proliferation induced by mitogens (Figure 3) (Findeisen et al., 2011). The suppression in the activity of Classes I and II HDACs by apicidin inhibited proliferation in newborn pulmonary arterial SMCs and cell cycle arrest at G1 phase (Zhao et al., 2020). HDAC inhibition by butyrate abrogated Akt activation and subsequent downstream Akt targets, thereby promoting proliferation arrest (Zhao et al., 2020). Among SMCs, vascular smooth muscle cells (VSMCs) are crucial in regulating blood pressure and tissue repair (Jaminon et al., 2019). HDACs mediate the functions of VSMCs. SIRT1 could regulate VSMC proliferation and motility and induce cell cycle arrest at G1/S transition (Wang and Chen, 2020). Overexpression of the unspliced form of HDAC7 (HDAC7u) attenuated SMC proliferation by decreasing cyclin D1, whereas the spliced HDAC7 did not result in the same effect (Zhou et al., 2011a). HDAC7u showed binding with beta-catenin and inhibited its nuclear translocation; moreover, it mitigated beta-catenin activity (Zhou et al., 2011b). Knockdown of HDAC7 exacerbated neointimal formation in femoral artery wire injury animal model, indicating a potential therapeutic in AS (Margariti et al., 2009).

In addition, VSMC migration is tightly related to vascular remodeling, which is likely to trigger AS progression (Lin et al., 2021). Mechanical cyclic strain reportedly suppressed the migration of SMCs along with the elevation of acetylated histone H3 and HDAC7 and decreased the level of HDAC3/4 (Figure 2) (Yan et al., 2009). Treatment with HDAC inhibitor tributyrin diminished VSMC migration and reduced the level of HDAC7 (Yan et al., 2009).

Also, the formation and the accumulation of lipid-loaded foam cells is the critical step in the pathophysiology of AS in the vascular wall (Linton et al., 2000). The uptake of modified lipoproteins in macrophage, such as oxidized LDL and native and modified LDL, drives macrophages to form foam cells (Linton et al., 2000). Recent studies also discovered that SMCs can also form foam cells. The inhibition of HDACs is involved in the formation of foam cells (Figure 3). MS‐275, which is known as a specific class I HDAC inhibitor, inhibits human foam cell formation (Jeanblanc et al., 2015).

The inversion of macrophage to foam cells plays a pro-atherosclerotic role in the AS process (Moore et al., 2013). The macrophage uptake of lipoproteins depends on scavenger receptors (SRs), including SR-A, CD36, and lectin-like oxidized LDL receptor-1 (LOX-1) (Chistiakov et al., 2016). The inhibition of SRs suppresses the ingestion of lipoproteins and hinders the AS process. Schaeffer et al. revealed that LOX-1 expression could be stimulated by pro-inflammatory cytokines in macrophage, which elevated the uptake of ox-LDL by nidus macrophages (Pirillo et al., 2013). The deacetylation of RelA/p65 by SIRT1 induced the suppression of NF-κB signaling pathway and LOX-1 in macrophage, diminished the uptake of oxLDL and foam cell formation, and subsequently reduced the risk of AS (Stein et al., 2010). Moreover, resveratrol decreased the uptake of oxLDL and protected from AS (Ou et al., 2006).

### HDACs in Modulating the Function of Monocyte/Macrophage

Monocytes and macrophages are critical driving factors in the inflammatory disease process (Yang et al., 2014). They play an important role in the pathogenesis and the initiation of AS, because AS is lipid-driven and occurs with chronic inflammation (Naruszewicz, 1989). Monocytes and macrophages develop foam cells and pro-inflammatory phenotypes in response to oxidized LDLs (Adamson and Leitinger, 2011). Perturbation of this phenotype would bring beneficial outcomes in the management of the disease. An increasing amount of evidence pointed out that histone acetylation plays an important role in the modulation in monocytes and macrophages (Das Gupta et al., 2016). In particular, H3 acetylation plays an important role in macrophage phenotypic gene expression (Figure 2) (Zubair et al., 2021). Among the HDACs, HDAC3 acts as a key modulator in M1 macrophage polarization, thereby blocking M2 macrophage polarization (Figure 2) (Wang et al., 2015b). HDAC3 also inhibits NF-κB signaling by deacetylating NF-κB p65 subunit and inducing its association with the IκB-α (Rajendrasozhan et al., 2008). Moreover, HDAC3 is responsible for the modulation of lipopolysaccharide-induced M1 macrophage-associated inflammatory gene expression (Treuter et al., 2017). HDAC5 is also a modulator of inflammation in macrophages (Zhao et al., 2019).

Translocation of circulating monocytes to the artery wall is one of the prominent phenotypes in AS in endothelial dysfunction and lipoprotein retention (Manduteanu and Simionescu, 2012). Upon differentiation into macrophage, these cells play an important role in sustaining lipid homeostasis in the vessel wall and inflammatory mediator secretion. Lipoprotein uptake by macrophage in the initiation of plaque contributes to the formation of lipid-load macrophage foam cells, which are hallmarks of AS (Remmerie and Scott, 2018). These foam cells stick to the artery walls, resulting in adverse inflammatory response that induces the recruitment and activation of other immune cells. Thus, chronic inflammation is maintained, which stimulates the progression of plaque formation. The role of HDACs in this process should not be ignored.

Histone acetylation also plays an important role in the cholesterol metabolism of macrophages (Zubair et al., 2021). Alterations in SIRT1 and SIRT6 promote cholesterol efflux by activating ABCA-1 and ATP-binding cassette subfamily G member (ABCG-1), leading to reduced macrophage-derived foam cell formation (D'Onofrio et al., 2018). During AS, histone modification within the atherosclerotic plaque affects macrophage phenotype; acetylation level alteration on H3K9 and H3K27 has been detected from human advanced AS plaques (Zhu et al., 2021).

*LDLR* ^−/−^ mice, as a general AS mice model, were fed with atherogenic diet and exhibited elevated HDAC3 and HDAC9 expressions during monocyte differentiation to macrophages (Figure 3) (Davis and Gallagher, 2019). HDAC3/9 systemic or myeloid-specific depletion reduced AS by increasing M2 macrophage polarization and lowering proinflammation gene expression (Sanchez-Lopez et al., 2019). Genome-wide association studies depicted that *HDAC9* genetic variants were associated with coronary artery disease and AS (Prestel et al., 2019). Systemic and bone marrow-specific depletion of *HDAC9* brought about elevated lipid homeostatic genes, reduced inflammatory genes, and switched macrophage phenotype to the M2 state, thereby decreasing AS progression by increasing the acetylation of the promoter of ABCA-1 and ABCG-1 in macrophages (Cao et al., 2014). Simultaneously, HDAC3 depletion induced macrophage phenotype switch, increased anti-inflammatory cytokine secretion, and reduced pro-inflammatory cytokine, thereby suggesting the promotion effect of HDAC3 on AS. The above mentioned HDACs mediated the recruitment and differentiation of monocytes and modulated AS progression (Hoeksema et al., 2014). However, the underlying mechanism of HDAC-mediated monocyte differentiation remains unclear and needs future research.

### The Functions of HDACs in the Pathogenesis of Atherosclerosis

AS is featured by the accumulated lipid and fatty streak lesion formation in the vessel, acting as the most prevalent disease in vasculature (Mundi et al., 2018). Notably, the recruitment of circulating monocytes to the arterial wall and lipoprotein retention is one of the earliest events of AS (Moore et al., 2013). The differentiation of monocytes to macrophages sustains the homeostasis of lipid in the vessel wall and secretion of inflammatory mediators, thereby functioning critically in the pathophysiology of AS (Rajendrasozhan et al., 2008). Macrophage lipid uptake in the initiation of plaque leads to the formation of lipid-loaded foam cells, which are hallmarks of AS (Rajendrasozhan et al., 2008). The presence of these foam cells at the artery wall stimulates disadvantageous immune response, which further leads to the recruitment and activation of other immune cells, maintains chronical immune response, and induces plaque progression (Wilson, 2010). Epigenetic modification in VSMCs (the dominant cell type in the arterial wall), such as acetylation, is reportedly associated with AS formation.

**FIGURE 4:**
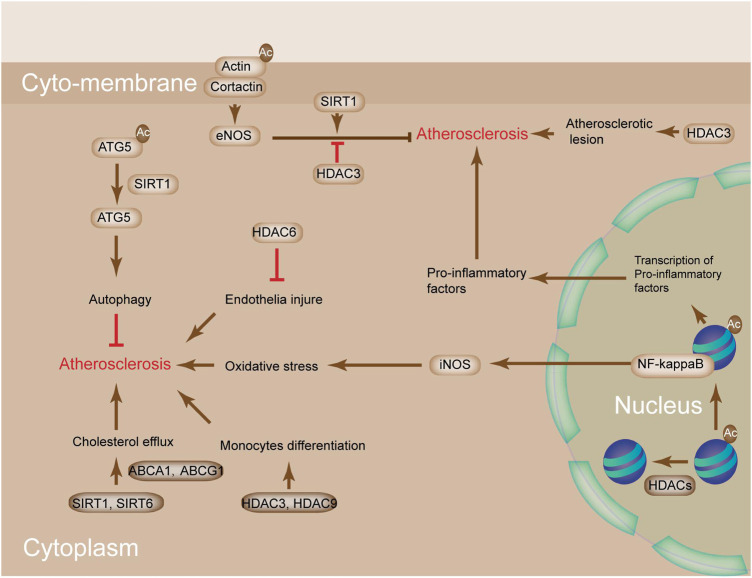
The potential mechanism of HDACs in atherosclerosis. SIRT1 and SIRT6 perturbation promotes cholesterol efflux by activating ABCA1 and ABCG1, leading to reduced macrophage-derived foam cell formation. Deacetylation of ATG5 by SIRT1 activates ATG5 to increase autophagy, which protects from AS. SIRT1-mediated deacetylation of cortactin promotes the translocation of cortactin to the cell periphery, whereas its interaction with cortical actin activates eNOS under shear stress conditions, which increases the bioavailability of nitric oxide (NO) and protects from AS. HDAC3/9 are stimulated in response to monocyte differentiation to macrophages in *LDLR* ^−/−^ mice kept on an atherogenic diet. HDAC6 is implicated in the prevention of endothelial injury and AS. NO bioavailability is increased by SIRT1-mediated deacetylation of eNOS and decreased by HDAC3-mediated deacetylation of eNOS. Acetylation of NF-κB subunits activates the expressions of Nos1, Nos2, and pro-inflammatory genes. High levels of NO and oxidative stress promote AS.

The effect of cholesterol metabolism on AS is obvious. The metabolic homeostasis of cholesterol includes cholesterol uptake, synthesis, and efflux and is critical in maintaining the homeostasis of blood vessel (Ghosh, 2011). Histone acetylation, which is affected by HDACs, is important in cholesterol metabolism. For instance, perturbations in SIRT1 and SIRT6 facilitate cholesterol efflux by activating ABCA-1 and ABCG-1, thereby leading to the reduced formation of macrophage-derived foam cells (Figure 4) (Ghosh, 2011). Apart from this, the alteration of histone acetylation by HDACs also affects macrophage phenotype in AS plaque. The alteration of acetylation on H3K9 and H3K27 is observed in advanced plaques compared with healthy samples (Lee et al., 2020). The levels of several HDAC, HDAC3, and HDAC9 could be stimulated in response to monocyte differentiation to macrophages in LDLR^−/−^ mice kept on an atherogenic diet (Figure 4) (Rajendrasozhan et al., 2008). In addition, myeloid-specific deletion of HDAC9 and HDAC3 mitigates AS by promoting M2 macrophage polarization and reducing proinflammatory gene expression (Qiu et al., 2021). The mechanism of HDAC9 in promoting pro-inflammatory responses and augmenting the effects of atherosclerotic plaque vulnerability involves the binding of HDAC9 to IKKα and β, which contributes to their deacetylation and subsequent activation, thereby finally driving vascular inflammation (Van den Bossche et al., 2014).

In addition to cholesterol metabolism, several biological processes are involved in the AS process, including the following: endothelial denudation, injury, activation, and shear stress; local platelets adherence; lipoprotein oxidation; lipoprotein aggregation; and inflammatory cytokine secretion and foam cell formation (Luchetti et al., 2017). The levels of Class I, IIa, IIb, and IV HDAC isoenzymes were dramatically augmented in human and mice AS tissues (Lian et al., 2020). Treatment with SAHA, a HDAC non-selective inhibitor, mitigated the level of atherosclerotic injury and reduced the production of ROS induced by NAPDH and pro-inflammation markers (Zhao et al., 2020). HDAC1 was suppressed in atherosclerotic lesions and aortic EC-treated with oxidized lipoproteins (Chen et al., 2020). Arginase 2 (Arg2) regulates the generation of endothelial nitric oxide, proliferation, fibrosis, and inflammation, making it a possible target for AS progression (Yang and Ming, 2014). Arg2 ablation by HDAC2 overexpression in human aortic ECs impaired EC activation induced by oxLDL (Pandey et al., 2014). Shear stress in the vicinity of disturbed flow could upregulate the expression of HDAC3. HDAC3 depletion aggravated atherosclerotic lesion in aortic isografts of *ApoE*-knockout mice, thereby indicating the protective role of HDAC3 in AS (Zhao et al., 2020).

HDACs are also involved in the modulation of cholesterol efflux (Cruz et al., 2021). The inhibition of HDACs upregulates the expression of cholesterol efflux genes *ABCA1* and *ABCG1*. Perturbations in SIRT1 and SIRT6 facilitate cholesterol efflux by activating ABCA-1 and ABCG-1, leading to the reduced formation of macrophage-derived foam cells (Sosnowska et al., 2017). Deacetylation of autophagy protein 5 (ATG5) by SIRT1 activates ATG5 to increase autophagy, which protects from atherosclerosis (Jiang et al., 2016). Furthermore, HDAC3 remains the only HDAC that is upregulated in human atherosclerotic lesions and accompanied by inflammatory macrophages, thereby indicating that it could be a potential target in AS prevention. Macrophage-induced inflammatory response and SMC-elicited vascular remodeling are the two main pathophysiological features in AS (Deeb and Hajjar, 2016). CIITA, known as a major histocompatibility class II transactivator, is a key modulator in these processes. It modifies IFN-gamma-induced major histocompatibility class II activation and inhibits type I collagen (Wu et al., 2009). HDAC2 counteracts CIITA activation via the degradation of CIITA, which is dependent on deacetylation activity (Kong et al., 2009). HDAC6 activity was greatly induced in *ApoE* ^−/-^ mice fed on high-fat diet in spite of the unchanged protein level, thereby indicating that HDAC6 inhibition can prevent endothelial injury and AS (Xu et al., 2021). The phosphorylation of HDAC5 was induced in A10 vascular SMCs in response to IGF-1 (Truong et al., 2021). Interference of IGF-1 receptor tyrosine kinase and NAD(P)H oxidase mitigated IGF-1 induced HDAC5 phosphorylation, thereby suggesting that HDAC5 phosphorylation is related to NAD(P)H oxidase-induced ROS generation and vascular disorders (Zhao et al., 2020).

Inflammation is considered as a main inducer of AS. Histone H3 acetylation mediated by the type A KAT p300 transcription factor is a prerequisite for the expression of inflammatory genes in the VSMCs of rats (Sun et al., 2017). Furthermore, treatment with HDAC inhibitor trichostatin A promotes the expression of inflammatory cytokine tumor necrosis factor (TNF) and aggravates the development of neointima lesions in AS-susceptible *LDLR* ^−/-^ mice (Okamoto et al., 2006). Histone acetylation is important in AS progression, and the inflammatory status is modulated by HDAC activity (Cosío et al., 2004). Vascular injury decreases the expression of VSMC differentiation marker genes and transforms it into a more proliferative phenotype, thereby increasing the probability of AS (Morris et al., 2019). Promoting the differentiation of VSMCs by histone H4 acetylation can restore injury in response to vascular stress by assisting the combination of serum response factor (SRF) and myocardin with CArG elements (Xiao et al., 2012). This process can be reversed by Kruppel-like factor 4 (KLF4), which promotes the deacetylation of histone H4 by recruiting HDAC2 (McDonald et al., 2006). SIRT1 exerts its anti-atherosclerotic effect by deacetylating autophagy protein 5, thereby impeding the oxLDL-induced cytotoxicity. The inhibition of SIRT1 facilitated the development of atherosclerotic plaque formation in *ApoE* ^−/−^ mice (Yuan et al., 2020). Other studies also showed that SIRT1 might prevent atherosclerosis by modulating eNOS activation. In human umbilical vein endothelial cells (HUVECs), cortactin phosphorylation by AMPK, deacetylation by SIRT1, and eNOS deacetylation by SIRT1 had atheroprotective effects (Figure 4) (Shentu et al., 2016), whereas eNOS deacetylation by HDAC3 promoted AS. Different from the reduced level of eNOS in the atherosclerosis state, neuronal NO synthase (nNOS) levels were induced in neointimal and media VSMCs (Nakata et al., 2007). Inducible NO synthase (iNOS) was also induced in atherosclerotic situations and associated with oxidative stress and inflammation (Förstermann and Sessa, 2012). Nuclear factor-κB (NF-κB) is responsible for the induction of nNOS in VSMC and iNOS and pro-inflammatory genes in ECs. The transcriptional factor NF-κB is modulated by lysine acetylation. SIRT1 deacetylation on p65 destroys the interaction of p300 and NF-κB, thereby reducing NF-κB transcriptional activity (Figure 4) (Li et al., 2021). SIRT1 is modulated by iNOS levels through increase in the production of NO, activity of NF-κB, and expression of pro-inflammatory genes (Lee et al., 2009). These studies indicated that HDACs offered protected against AS.

The diverse functions of HDACs in AS have been mentioned above (Table 1). However, the role of HDACs in AS often leads to conflicting results. The overexpression of HDAC3, HDAC5, and HDAC7 displayed a pro-atherosclerotic feature (Zhang et al., 2018b). HDAC3 depletion induced the increase in the expressions of IL-4-activated genes and activated anti-inflammatory phenotype due to the reprogramming-like effect on macrophage (Kuznetsova et al., 2020). Also, the HDAC9 ablation in mice repressed the expression of inflammatory-related genes and cytokine secretion in macrophage in response to LPS (Liu et al., 2021). However, HDACs seemed to exhibit atherosclerosis protection in animal models (Halili et al., 2010). HDAC3 depletion in ECs facilitated the increase in neointimal formation, thereby implicating the beneficial role of HDAC3 in AS. Similarly, HDAC inhibitor treatment, such as that of Trichostatin A, by intraperitoneal injection resulted in augmented plaque size and macrophage infiltration in plaques in *LDLR* ^−/-^ mice (Manea et al., 2020). These findings suggested the complicated effects of HDACs on AS and indicated that HDACs in a specific cell type affect different phenotypes in atherogenic progression.

**TABLE 1:** Roles of HDACs in blood vessels and the pathology of AS.

Subtypes	Classification	Functions and Phenotypes
HDAC1	Class I HDACs	Stimulates angiogenesis, Promote cell survival and prevent cell apoptosis
HDAC2		Inhibit cell proliferation, Inhibit vascular dysfunction
HDAC3		Stimulate EC differentiation, Upregulate in atherosclerosis
HDAC8		The marker of smooth muscle differentiation
HDAC4	Class IIa HDACs	Contributes to angiogenesis
HDAC5		Repress angiogenesis
HDAC7		Stimulate cell migration, Rupture of blood vessels
HDAC9		Increase EC permeability, Develop atherosclerosis
HDAC6	Class IIb HDACs	Increase blood pressure and vasoconstriction, increase vascular hyperplasia or vasoconstriction, Develop atherosclerosis
HDAC10		Stimulate tube formation
HDAC11	Class IV HDACs	Induce vessel injury
SIRT1	Class III HDACs	Modulate homeostasis, Inhibit vascular remodeling, Inhibit neointima formation, Enhance EC survival, Anti-inflammation
SIRT3		Decrease proliferation
SIRT6		Prevent senescence

Despite the diverse effects of HDACs in AS pathogenesis (Table 1), some issues need to be discussed. One question to address is whether the effects of HDACs on tissues or cell types are specific. Another problem is whether or not the effects of HDACs on the migration of SMCs and ECs are the same. To answer these questions, more experiments are required in future.

### Promising Therapeutic Targets in AS

HDACs are involved in broad biological processes. Thus, multiple HDAC inhibitors are developed to target the catalytic domain in HDACs (Table 2). The inhibitors can be classified into four groups, namely, hydroxamic acids, short chain fatty acids, cyclic tetrapeptides, and benzamides, based on their structural diversity (Table 2) (Li and Seto, 2016).

**TABLE 2:** Effects of HDACis in AS.

HDAC Inhibitor	Type	HDAC Specificity	Effects in AS
Valproic acid	Short-chain fatty acids	Class I, IIa	Long-term treatment promotes angiogenesis
Sodium butyrate	Short-chain fatty acids	Class I, II	Impairs atherogenesis
Trichostatin A	Hydroxamic acid	Class I, II, IV	Promotes atherosclerosis, Short-term treatment reduces angiogenesis, Long-term treatment promotes angiogenesis
Vorinostat/SAHA	Hydroxamic acid	Class I, II, IV	Short-term treatment reduces angiogenesis
Tubastatin A	Hydroxamic acid	HDAC6	Alleviates Ang II-induced vasoconstriction, decreased the intimal VSMC proliferation and neointimal hyperplasia
Entinostat/MS275	Benzamide	Class I	
Mocetinostat	Benzamide	Class I, IV	
Apicidin	Cyclic peptide	Class I	Increases vessel calcification
Romidepsin	Cyclic peptide	Class I	
Cambinol	SIRT inhibitor	SIRT1, SIRT2	Reduces inflammation
Nicotinamide	SIRT inhibitor	Class III (SIRTs)	
Metacept-1	Synthetic derivate of oxamflavin		Inhibits MMP-2 expression in VSMC
Apicidin	Cyclic peptides	Class I	Inhibits TF activity and protein level

Inhibitors of HDACs generally cause growth arrest, cellular differentiation, and apoptosis. Thus, they are used for cancer treatment (Li and Seto, 2016). The HDAC non-selective inhibitors, SAHA/vorinostat and Romidepsin (Istodax, FK228), which are characterized by a relatively low IC50 for HDAC1/2, have been approved for the clinical treatment of cutaneous T cell lymphoma by FDA in the United States (Bertino and Otterson, 2011). Romidepsin facilitates the acetylation of non-histone substrates involved in the transcription of VCAM-1 in ECs, which mediates the AS process (Tambaro et al., 2010). In addition, Romidepsin treatment in *Apoe* ^−/-^ mice protected from diet-induced atherosclerotic lesion accumulation (Nicorescu et al., 2019). Given the elevated level of HDAC1/2 in advanced AS and the clinical availability of Romidepsin, the use and mechanism of this specific inhibitor in AS deserve further investigation. A potent HDAC inhibitor, suberoylanilide hydroxamic acid, exhibits anti-inflammatory properties by attenuating the LPS-induced expression of NF-κB-regulated cytokines (Zhao et al., 2015). Sodium valproate, a class I HDAC inhibitor, reportedly facilitated the phenotype switch of macrophage, thereby delaying AS progression (Chen et al., 2014). Pharmacological inhibition of HDAC1/2/3 by scriptaid prevented smooth muscle cell proliferation and neointima formation (Findeisen et al., 2011).

Commonly used HDAC inhibitors in experimental animal models and clinical trials are the Class I and II HDACs. These consist of the natural products butyrate and Trichostatin A (TSA). Although the inhibition of HDACs in macrophage brought about beneficial effects to AS, the broad usage of these inhibitors is limited by the observation that TSA unexpectedly promoted the progression of plaque expansion in an AS mouse model (Choi et al., 2005). This result might be due to the negative effects of TSA on other cell types in AS, such as ECs and SMCs. Therefore, the inhibition of HDACs in macrophage and monocytes is beneficial in the AS state. Findeisen et al. reported that scriptaid, a non-selective HDAC inhibitor, protected from neointimal thickening both *in vitro* and *in vivo* in a mouse model (Findeisen et al., 2011). Also, they demonstrated that scriptaid had no obvious toxicity at the dosage used.

Treatment with TMP195, a selective inhibitor of Class IIa HDAC, suppressed critical inflammatory pathways and mitigated atherogenesis in advanced stage AS, thereby offering a novel therapeutic strategy for reducing the consequence of vascular inflammation (Asare et al., 2020). Another Class II HDAC selective inhibitor, MC1568, rescued serum-dependent histone acetylation in NO-induced HUVECs (Illi et al., 2008). HDAC3-specific inhibitor RGFP966 suppressed endothelial-to-mesenchymal transition by modulating inflammatory response in AS (Chen et al., 2021).

## Outlook

AS is a common pathological basis of cardiovascular and cerebrovascular diseases and seriously endangers human health. With the further understanding of the pathogenesis of AS, an increasing amount of evidence suggested the important role of HDACs in AS. The effects of HDACs on atherosclerosis are complex and multifaceted. In ECs, SMCs, and even macrophages, HDACs play different roles in regulating cell proliferation, migration, apoptosis, differentiation, inflammation, and oxidative stress (Zhou et al., 2011a; Grimaldi et al., 2015). It is difficult to characterize the overall effect of HDACs on AS. A growing number of recent studies suggested that HDACs can be used as potential therapeutic targets for AS (Neele et al., 2020; Wong et al., 2021). Inhibitors of HDACs can improve the symptoms of AS by precisely inhibiting the deacetylase activity of HDACs (Morrell et al., 2013). The development of inhibitors of HDACs is one of the current research hot spots. The current HDACi market, which includes existing drugs, is expected to expand into other indications, such as cardiovascular and cerebrovascular diseases (e.g., AS) (Bagchi and Weeks, 2019).

It is well known that the majority of existing or clinically available HDAC inhibitors are generic (Bondarev et al., 2021). The development of selective HDACi could reduce the side effects of other target activities, such as the potential generic toxicity of HDAC6 (Yang et al., 2017). However, as there are many subtypes of HDACs, and there are many similarities in the active domain and catalytic site among the subtypes, the development of HDAC inhibitors with high subtype selectivity is a breakthrough point that can be reached in the future and that will likely face great challenges in practical research. Clinical verification is needed to test the efficacy. In addition, the development of dual-target HDAC inhibitors is one of the current research directions (Jin et al., 2021). While remaining active against HDAC, they also act on one or more targets related to AS, which is worth studying in the future. Dual-target HDAC may be expanded and improved in terms of indications and efficacy to some extent, but problems (e.g., high toxicity of dual-target HDAC inhibition) exist (Peng et al., 2020; Kuznetsoff et al., 2021). These issues need to be addressed in future research works. HDACi has been extensively studied in recent years and has been regarded to have a potential therapeutic effect on many diseases, including neurodegenerative diseases, autoimmune diseases, acute graft-versus-host disease, and so on (Vojinovic et al., 2011; Ghiboub et al., 2021). Its potential therapeutic effect on AS is also worth exploring through further research. Other issues that need to be addressed are the targeting and specificity of HDACi. Researchers need to find out how to accurately make HDACi act on the AS region without changing the function of other normal cells. In future studies, more efficient and accurate HDAC inhibitors need to be developed, so AS to improve the therapeutic effect of related inhibitors on AS. With further research, more functions of HDACs in the pathogenesis of AS will be revealed, which will help us find a better plan to fight this disease.
